# Detection and differentiation of Schmallenberg, Akabane and Aino viruses by one-step multiplex reverse-transcriptase quantitative PCR assay

**DOI:** 10.1186/s12917-015-0582-7

**Published:** 2015-10-24

**Authors:** Ji-Hye Lee, Hyun-Ji Seo, Jee-Yong Park, Sung-Hee Kim, Yun Sang Cho, Yong-Joo Kim, In-Soo Cho, Hye-Young Jeoung

**Affiliations:** Animal and Plant Quarantine Agency, Anyang, Gyeonggi-do 430-824 Republic of Korea

**Keywords:** One-step mRT-qPCR, SBV, AKAV, AINV

## Abstract

**Background:**

Schmallenberg virus (SBV), Akabane virus (AKAV) and Aino virus (AINV) are members of the Simbu serogroup within the genus *Orthobunyavirus*, family *Bunyaviridae,* which can cause reproductive disorders including abortion, stillbirth and congenital malformation in ruminants. Because, the clinical signs are similar, confirmatory diagnosis requires viral detection to differentiate infection between these three viruses.

**Methods:**

In this study, a one-step multiplex reverse-transcriptase quantitative PCR (one-step mRT-qPCR) was developed for the simultaneous detection and differentiation of SBV, AKAV and AINV.

**Results:**

The detection limit of the one-step mRT-qPCR for SBV, AKAV and AINV were 2.4 copies (10 ^0.6^ TCID _50_/ml), 96.2 copies (10 ^1.5^ TCID _50_/ml) and 52.3 copies (10 ^1.2^ TCID _50_/ml), respectively. Various field samples such as bovine serum, bovine whole blood, bovine brain, goat serum and *Culicoides* were analyzed using the one-step mRT-qPCR and compared with previously published RT-qPCRs. The test results of the field samples were identical for the one-step mRT-qPCR and RT-qPCRs, which showed all samples to be negative for SBV, AKAV and AINV, except for one bovine brain sample (1/123) that was positive for AKAV.

**Conclusion:**

The one-step mRT-qPCR allows for the simultaneous detection of three viral pathogens (SBV, AKAV and AINV) that cause reproductive failure.

## Background

The bunyavirus genus consists of 18 serogroups, of which the simbu serogroup composed of 24 antigenically related arthoropod-borne viruses are present throughout the world [1, 2]. In particular, 2 Simbu serogroup bunyaviruses, the Akabane virus (AKAV) and Aino virus (AINV) have been frequently reported in Asia, including Republic of Korea (ROK) [3–7]. Since August 2011, a novel *Orthobunyavirus* named the Schmallenberg virus (SBV), which is closely related to simbu serogroup viruses, has been reported in ruminants such as cattle and sheep across Europe [8, 9]. These three bunyaviruses, SBV, AKAV and AINV are primarily transmitted by biting midges, and cause reproductive disorders including abortion, stillbirth and congenital malformation in ruminants resulting in considerable economic losses to the livestock industry [8, 10, 11]. Because, the clinical signs are so similar, confirmatory diagnosis requires viral detection in order to differentiate infection between these three viruses. This can be especially important if any of the viruses are exotic to a country, which would require rapid detection to quickly identify any new incursions. In ROK where AKAV and AINV are reported, a new introduction of SBV could be misdiagnosed for an endemic disease, possibly resulting in the rapid spread of SBV before it’s eventually identified. To avoid this scenario, testing for exotic diseases such as SBV should be included when diagnosing suspect cases of AKAV or AINV. However, most commercial diagnostic detection kits are only available for each of the viruses, and conducting single real-time PCR assays targeting individual viruses are expensive, labor-intensive and time-consuming. Thus, a cost-effective, convenient and rapid laboratory assay which allows simultaneous diagnosis of several viruses in clinical samples would be useful for such applications enabling rapid differentiation between endemic and exotic diseases that are clinically similar [12].

In this study, a one-step multiplex reverse-transcriptase quantitative PCR (one-step mRT-qPCR) was developed for the simultaneous detection and differentiation of SBV, AKAV and AINV. The test was applied to clinical samples collected from ROK such as bovine serum, bovine whole blood, bovine brain, goat serum and *Culicoides* biting midges for further evaluation.

## Results

### Reproducibility

To assess the analytical intra- and inter assay reproducibility of the amplification step of the assay, Cp values were compared between replicates of 10-fold serial dilutions tested in same batch or on different days. The coefficient of variation (CVs) was calculated to measure the inter- and intra-reproducibility of the assay. The CVs in intra assay ranged from 0.21 % to 1.02 %, and the CVs in inter assay ranged from 0.33 % to 1.78 %. The results showed good reproducibility.

### Sensitivity of one-step mRT-qPCR

**Fig. 1:**
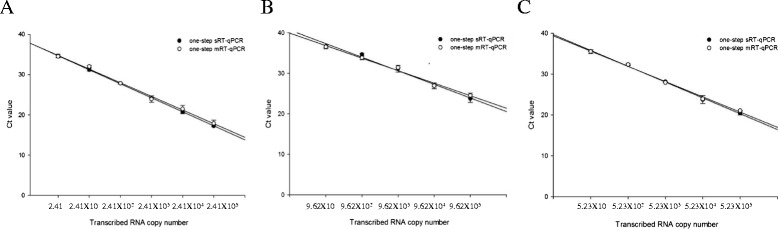
Analytical sensitivity of one-step sRT-qPCR and one-step mRT-qPCR. Serial dilutions of **a** in vitro transcribed SBV RNA, **b** in vitro transcribed AKAV RNA, and **c** in vitro transcribed AINV RNA, were amplified in one-step sRT-qPCR and one-step mRT-qPCR

Ten-fold serial dilutions of each of the in vitro transcribed RNAs or viral RNAs were tested, and repeated in triplicate. The Cp values and detection limit of the one-step mRT-qPCR were compared with the one-step single reverse-transcriptase quantitative PCR (one-step sRT-qPCR) format for each of the viruses using the same templates. There were no appreciable differences in the mean Cp values between the one-step sRT-qPCR and one-step mRT-qPCR (Fig. 1) (data of tested viral RNA not shown). The detection limit of the one-step mRT-qPCR were 2.41 copies (10 ^0.6^ TCID _50_/ml) for SBV, 96.2 (10 ^1.5^ TCID _50_/ml) for AKAV and 52.3 copies (10 ^1.2^ TCID _50_/ml) for AINV. The mRT-qPCR was also compared with previously published RT-qPCRs [13, 14] for each of the viruses using the same templates, which showed similar results. The detection limit of the published RT-qPCRs were 2.41 copies (10 ^0.6^ TCID _50_/ml) for SBV, 96.2 (10 ^1.5^ TCID _50_/ml) for AKAV and 523 copies (10 ^2.2^ TCID _50_/ml) for AINV. The detection limits for inter- and intra-assay and spiking assay were shown to be same as those of the one-step mRT-qPCR.

### Specificity of one-step mRT-qPCR

The test performance of the one-step mRT-PCR for transcribed RNAs or viral RNAs of the negative control viruses, which included bovine ephemeral fever virus, Chuzan virus, Ibaraki virus, West Nile fever virus NY99 stain, West Nile fever virus B956stain, Japanese encephalomyelitis virus and bluetongue virus, demonstrated neither non-specific reaction nor any inter-assay cross amplification as same results of one-step sRT-qPCR.

### Detection of three viruses in field samples

The performance of the one-step mRT-PCR assay was evaluated and compared with previously published RT-qPCRs [13, 14] using several different types of field samples collected in ROK, including bovine serum, bovine whole blood, bovine brain, goat serum, and *Culicoides* biting midges. No SBV and AINV were detected in any of the samples by any of the tests. AKAV was detected in one bovine brain sample (1/123) by both the one-step mRT-PCR and published RT-qPCR [14] (Table 1). The AKAV positive sample was further confirmed by additional RT-PCR tests [1], and by nucleotide sequencing.

**Table 1:** Detection of viruses in clinical samples using one-step mRT-qPCR

	Bovine			Goat serum	Culicoides
	Whole blood	Serum	Brain		
SBV^a^	0/112^d^	0/100	0/123	0/100	0/157
AKAV^b^	0/112	0/100	1/123	0/100	0/157
AINV^c^	0/112	0/100	0/123	0/100	0/157

^a^Schmallenberg virus

^b^Akabane virus

^c^Aino virus

^d^Number of positive samples for each target virus /number of clinical samples

## Discussion

SBV, AKAV and AINV are responsible for reproductive failure in ruminants, which cause significant economic losses for farms [15–17]. Because these viruses can cause similar clinical signs associated with reproductive failure, there is a need for a sensitive and accurate diagnostic method that can simultaneously detect and differentiate infection from these viruses.

In a few short years, SBV has quickly become established in many countries in Europe, and due to increased international trade and travel, a new introduction of SBV into previously free countries cannot be ruled out. Once introduced it could then quickly become established as was the case in Europe. Also, recent studies indicate that antibodies to AKAV and AINV will not provide protective immunity to SBV [18], and therefore it is likely that a new introduction of SBV would cause wide spread outbreaks even in countries where similar bunyaviral infection such as AKAV and AINV are already present. Such countries will benefit from a rapid diagnostic test that can quickly identify and also differentiate SBV from AKAV and AINV. The ROK is one such country, which similar to many countries in Asia, has had many reported incidences of AKAV and AINV detection [3, 4], particularly AKAV [19, 20].

Various RT-PCR and qPCR assays have been published for the detection of SBV, AKAV or AINV [1, 13, 14]. But, simultaneous detection and differentiation of SBV, AKAV and AINV in a RT-qPCR format has not been previously reported. In this study, a one-step mRT-qPCR was developed for the simultaneous detection and differentiation of Schmallenberg virus (SBV), Akabane virus (AKAV) and Aino virus (AINV). When compared to previously published RT-qPCRs, the one-step mRT-qPCR provided comparable level of sensitivity and specificity. The one-step mRT-qPCR was further evaluated using various clinical samples, such as bovine serum, whole blood and brain tissue, goat serum, and *Culicoides*. The samples used for the evaluation were selected as the three viruses have been previously detected in these clinical samples, except for serum [13, 21–23] which has only been reported for SBV and AKAV [1, 21]. Also, *culicoides* were selected as they are regarded as being the major vectors for all three viruses [24, 25]. In this study, only AKAV was detected in one bovine brain sample. However, this could be due to all clinical samples, except for the brain samples, being collected from healthy cattle and goats at farms or slaughter, and short duration of viraemia for AKAV and AINV in blood [1].

## Conclusion

A one-step mRT-qPCR assay was developed to provide a rapid and sensitive diagnostic method for the simultaneous detection and differentiation of three viral pathogens associated with reproductive failure in clinical samples. The assay will be a useful tool for countries with AKAV and AINV, but is free from SBV or vice versa, to conduct diagnosis not only for suspect diseases in the country, but for diseases that is exotic to the country that would need to be quickly identified if any incursion was to occur.

## Methods

### Viruses

Schmallenberg virus, Akabane virus 93FMX strain (KVCC-VR0000064), and Aino virus KSA9910 (KVCC-VR0000064) were maintained in Vero cells. All cell lines were grown in Dulbecco’s modified Eagle’s medium (GibcoBRL, Gaithersburg, MD, U.S.A) supplemented with 5 % heat-inactivated fetal bovine serum (GibcoBRL) in a humidified 5 % CO_2_ atmosphere at 37 °C. Schmallenberg virus was kindly provided by Friedrich Loeffler Institute (FLI). The Akabane virus and Aino virus were acquired from the Korea veterinary Culture Collection (KVCC) .

### RNA extraction

*Culicoides* samples were pooled (30–40) and added to 2 ml containers with ceramic beads. Samples were ground for 30 s with 1 ml of cold phosphate-buffered saline (PBS). Ground samples were centrifuged (1 min, 4 °C), and supernatants were harvested. Total viral RNA was extracted from each control viruses, blood samples, serum samples, bovine brain samples, and pooled *culicoides* samples using the Maxwell^®^16 research instrument system (Promega, Medison, Wisconsin, USA) with Maxwell^®^16 viral total nucleic acid purification kit (Promega AS1150), according to the manufacturer’s instructions.

### Specific primer and probe design

Oligonucleotide primers and probes were used to amplify the genes encoding the S segment of SBV (Accession No: HE649914), AKAV (Accession No: AF034942) and AINV (Accession No: AF034939). These conserved viral genome regions were chosen as the best candidates for the generation of specific primers and virus-specific probe sequences for SBV, AKAV and AINV. The primer and probe sequences, fluorophores and quenchers as follows: Primer Common F 5’ – TGACTGCAGAAGARTGGATGA-3’, Common R 5’ – GAATCCA GATTTGGCCCA -3’, SBV Probe 5’ – FAM-ACAGAAATAAAAGCTGCT-BHQ1-3’, AKAV Probe 5’ – HEX-ATCTAAGTTGGACGCA-BHQ1-3’, and, AINV Probe 5’ – Cy5-A TGCTGTCCGTGCA-BHQ2-3’.

### Preparation of RNA controls

Plasmids containing target sequences of the mRT-qPCR for SBV, AKAV and AINV were produced using the pGEM®-T Easy Vector Systems (Promega), and RNA was transcribed using the MEGAscript® Kit (Ambion) according to manufacturer’s instructions. RNA quality and integrity was confirmed using Nanodrop 2000 (Thermo scientific), and stored at −20 °C.

### One-step sRT-qPCR and one-step mRT-qPCR

The one-step sRT-qPCR reaction was used to detect each control RNAs or viral RNA using specific primer, probe and viral RNA. The one-step mRT-qPCR reaction contained all three control RNAs or viral RNAs, a mixture of all three primer pairs and probe in the same tube. The one-step sRT-qPCR and one-step mRT-qPCR were tested using AgPath-ID One-Step RT-PCR Kit (Applied Biosystems). The reaction contained 12.5 μl 2x RT-PCR buffer, 1 μl 25x RT-PCR enzyme mix, 5 μl RNA template, 5 μl primer-probe mix (Final concentration of 3.2 μM for each primer and 200nM for each probe) and RNase free water to final volume of 25 μl. All reaction were performed on a BioRad CFX96 with the following cycling parameters; 45 °C for 10 min, 95 °C for 10 min and then 45 cycles of 95 °C for 15 s 52 °C for 20s. The primer and probe concentrations for each assay were individually optimized using in-house protocol. Each control RNAs and viral RNAs were quantified using Nanodrop 2000 (Thermo scientific).

### Sensitivity of one-step sRT-qPCR and one-step mRT-qPCR

The sensitivity of one-step sRT-qPCR and one-step mRT-qPCR was evaluated on serial ten-fold dilutions of control RNA or viral RNA. For spiking assay, serial ten-fold dilutions of the control RNAs were used to spike each of the matrix nucleic acid extracted from the ten samples (e.g. Bovine whole blood, serum and brain, Goat serum, and *Culicoides*). Also, serial ten-fold dilutions of the viruses were used to spike each of the ten samples (e.g. Bovine whole blood, serum, brain, Goat serum, *Culicoides*), from which RNA was extracted using Maxwell^®^16 viral total nucleic acid purification kit (Promega AS1150). The matrix nucleic acid and the samples used for the spiking assay were all tested negative against SBV, AKAV, and AINV, prior to use. Ten-fold serial dilutions of the control RNA or viral RNA were prepared from starting solutions containing 2.4 × 10^6^ copies (10 ^5.6^ TCID _50_/ml) for SBV, 9.62 × 10^6^ copies (10 ^5.5^ TCID _50_/ml) for AKAV, and 5.23 × 10^6^ copies (10 ^5.2^ TCID _50_/ml) for AINV.

### Inter assay and intra assay

Reproducibility of the Inter- and Intra-assay were examined in triplicate using ten-fold serial dilutions of the three control RNAs (starting solutions containing 2.4 × 10^6^ copies for SBV, 9.62 × 10^6^ copies for AKAV, and 5.23 × 10^6^ copies for AINV).

### Specificity of one-step sRT-qPCR and one-step mRT-qPCR

Bovine ephemeral fever virus (10 ^4.1^ TCID _50_/ml, KVCC-VR1300041), Chuzan virus (10 ^3.2^ TCID _50_/ml, KVCC-VR000109), Ibaraki virus (10 ^4.6^ TCID _50_/ml, KVCC-VR000109), West Nile fever virus NY99 stain (10 ^6.1^ TCID _50_/ml) West Nile fever virus B956 strain (10 ^6.7^ TCID _50_/ml), Japanese encephalomyelitis virus (Anyang strain, 10 ^7.1^ TCID _50_/ml, KVCC-VR1200026) and bluetongue virus (10 ^4.5^ TCID _50_/ml, RSArrr001). West Nile fever viruses were acquired from ATCC and bluetongue viruses were kindly provided by the Pirbright Institute. The other viruses were received from the Korean Veterinary Culture Collection (KVCC). Each probe was tested against three control RNAs and the viral RNA.

### Clinical samples

Bovine whole blood samples (n = 112) were collected at abattoir in Jeju island in 2013. Bovine and goat serum samples (n = 100) were collected with 20 samples being collected from national surveillance of Foot and Mouth disease by the Animal and plant Quarantine Agency (QIA) from each 5 farms located in various provinces. Bovine brain samples (n = 123) initially were submitted for etiological findings to the Animal and plant Quarantine Agency (QIA), *Culicoides* samples (157 pools) of various species were collected through the national vector surveillance program for arboviral infectious disease in collaboration with local entomological experts [26]. Samples were maintained at −70 °C until used. The collected clinical samples were tested by one-step mRT-qPCR and real-time reverse-transcriptase PCR for AKAV and AINV, and real-time reverse-transcriptase PCR for SBV as described previously [1, 13].

## Abbreviations

SBV: Schmallenberg virus
one-step mRT-qPCR: one-step multiplex reverse-transcriptase quantitative PCR
AKAV: Akabane virus
AINV: Aino virus
ROK: Republic of Korea
one-step sRT-qPCR: one-step single reverse-transcriptase quantitative PCR
FLI: Friedrich Loeffler Institute
KVCC: Korea veterinary Culture Collection
QIA: Animal and plant Quarantine Agency
